# Highly contiguous genomes of human clinical isolates of *Giardia duodenalis* reveal assemblage- and sub-assemblage-specific presence–absence variation in protein-coding genes

**DOI:** 10.1099/mgen.0.000963

**Published:** 2023-03-28

**Authors:** Christian Klotz, Marc William Schmid, Katja Winter, Ralf Ignatius, Filip Weisz, Christina Skar Saghaug, Nina Langeland, Scott Dawson, Marco Lalle, Kurt Hanevik, Simone M. Cacciò, Toni Aebischer

**Affiliations:** ^1^​ Department of Infectious Diseases, Unit 16 Mycotic and Parasitic Agents and Mycobacteria, Robert Koch-Institute, Berlin, Germany; ^2^​ MWSchmid GmbH, Hauptstrasse 34, 8750 Glarus, Switzerland; ^3^​ Bioinformatics Core Facility (MF1), Robert Koch-Institute, Berlin, Germany; ^4^​ MVZ Labor 28, Mecklenburgische Str. 28, 14197 Berlin, Germany; and Institute of Microbiology, Infectious Diseases and Immunology, Charité-University Medicine Berlin, Campus Benjamin Franklin, Hindenburgdamm 30, 12200 Berlin, Germany; ^5^​ Institute of Immunology and Microbiology, First Faculty of Medicine, Charles University, Prague, Czech Republic; ^6^​ Department of Clinical Science, University of Bergen, Bergen, Norway and Department of Medicine, Haukeland University Hospital, Bergen, Norway; ^7^​ Department of Microbiology and Molecular Genetics, UC Davis, Davis, CA, USA; ^8^​ European Reference Laboratory for Parasites, Department of Infectious Diseases, Istituto Superiore di Sanità, Rome, Italy

**Keywords:** allelic sequence heterozygosity, comparative genomics, *Giardia duodenalis*, protozoa

## Abstract

*Giardia duodenalis* (syn. *G. intestinalis, G. lamblia*) is a widespread gastrointestinal protozoan parasite with debated taxonomic status. Currently, eight distinct genetic sub-groups, termed assemblages A–H, are defined based on a few genetic markers. Assemblages A and B may represent distinct species and are both of human public health relevance. Genomic studies are scarce and the few reference genomes available, in particular for assemblage B, are insufficient for adequate comparative genomics. Here, by combining long- and short-read sequences generated by PacBio and Illumina sequencing technologies, we provide nine annotated genome sequences for reference from new clinical isolates (four assemblage A and five assemblage B parasite isolates). Isolates chosen represent the currently accepted classification of sub-assemblages AI, AII, BIII and BIV. Synteny over the whole genome was generally high, but we report chromosome-level translocations as a feature that distinguishes assemblage A from B parasites. Orthologue gene group analysis was used to define gene content differences between assemblage A and B and to contribute a gene-set-based operational definition of respective taxonomic units. *Giardia* is tetraploid, and high allelic sequence heterogeneity (ASH) for assemblage B vs. assemblage A has been observed so far. Noteworthy, here we report an extremely low ASH (0.002%) for one of the assemblage B isolates (a value even lower than the reference assemblage A isolate WB-C6). This challenges the view of low ASH being a notable feature that distinguishes assemblage A from B parasites, and low ASH allowed assembly of the most contiguous assemblage B genome currently available for reference. In conclusion, the description of nine highly contiguous genome assemblies of new isolates of *G. duodenalis* assemblage A and B adds to our understanding of the genomics and species population structure of this widespread zoonotic parasite.

## Data Summary

Raw reads produced within this project are deposited at the NCBI SRA database under BioProject accession number PRJNA879307 and available at https://www.ncbi.nlm.nih.gov/sra/PRJNA879307, accession numbers: SRR21529651, SRR21529650, SRR21529639, SRR21529628, SRR21529625, SRR21529624, SRR21529623, SRR21529622, SRR21529621, SRR21529620, SRR21529649, SRR21529648, SRR21529647, SRR21529646, SRR21529645, SRR21529644, SRR21529643, SRR21529642, SRR21529641, SRR21529640, SRR21529638, SRR21529637, SRR21529636, SRR21529635, SRR21529634, SRR21529633, SRR21529632, SRR21529631, SRR21529630, SRR21529629, SRR21529627 and SRR21529626. Software tools used in the study are publicly available and the sources have been provided within the article.

**Impact Statement**

*Giardia duodenalis* is a widespread protozoan parasite relevant for public health. The lack of high-quality genome assemblies for reference has hampered population genetic studies. Here, for reference we provide nine highly contiguous annotated genome assemblies of the two assemblage types A and B that derive from eight human clinical isolates and one cat isolate. Despite the high overall genomic synteny between assemblage A and B, there are specific genome rearrangements and orthologous gene groups that distinguish and classify the assemblages. Strikingly, the genome of one of the assemblage B isolates was characterized by an extremely low allelic sequence heterozygosity (0.002%), resulting in the most contiguous assemblage B genome described to date. Overall, the described genomes add a significant resource to the very few available *Giardia* genomes (and genomes of diplomonads in general) that can be exploited to illuminate the impact of genetic differences on poorly understood factors mediating disease manifestation or virulence, zoonotic potential and taxonomy.

## Introduction

*Giardia duodenalis* (syn. *G. intestinalis, G. lamblia*) is one of the most commonly diagnosed intestinal protozoan parasites worldwide and a significant public health concern. Transmission occurs via infectious cysts by the faecal–oral route, either directly by contact with another infected host (human or animal) or indirectly by uptake of cyst-contaminated food or water. Infection is often asymptomatic or causes variable symptoms such as diarrhoea, nausea and other unspecific gastrointestinal complaints. Infections are mostly self-limiting, but chronic infections are also observed [1–3]. It is unclear to what extent the variable course of disease is due to genetic differences between parasite isolates, including the presence, absence or polymorphism of specific virulence- or pathogenicity-associated genes [1].

Possible genotypic differences that overall distinguish distinct parts of the population of human-infecting *Giardia* parasites and their relevance as a basis for the parasites’ taxonomy also remain unresolved. Based on host range and molecular characteristics, *G. duodenalis* has been proposed to either represent a species complex of eight genetically distinguishable subgroups, called assemblages A–H [1], or to represent distinct biological species [4]. In the framing of the former classification, assemblages A and B are the aetiological agents of human giardiasis but are also found in a broad range of mammalian species and are therefore assigned zoonotic potential. Assemblages have been further subtyped into sub-assemblages (AI, AII and AIII; BIII and BIV), but standardized multiple locus sequence typing (MLST) schemes lack resolution to delineate all sub-assemblages reliably [1, 5–7]. Attempts to close this knowledge gap by sequencing and analysing more genomes are gaining momentum [8–22], but high-quality reference genome data remain sparse. Additional genomes, however, are needed to improve understanding of the genetic differences related to aspects such as disease manifestation and zoonotic potential and, more fundamentally, to resolve taxonomy.

*Giardia* parasites have two nuclei that are both diploid for their five chromosomes [21] but can display genomic plasticity resulting in uneven gene sets per nucleus [23]. Currently available data suggest a haploid genome size of ~12 Mb. Since the first *Giardia* genome sequence was published [14], only 13 further whole genome assembly data sets from axenic trophozoite isolates (as of July 2022) and often derived by Illumina short read technology have been deposited in GenBank [24]. Altogether, the data sets (often highly fragmented) include eight different ‘*Giardia intestinalis*’ lab isolates: three assemblage AI [WB/C6 (two different data sets), beaver and ZX15]; three assemblage AII (DH, AS98 and AS175); and two assemblage B [GS (three different data sets) and BAH15c1]. Each of these eight isolates was derived originally from humans. In addition, one genomic data set of a pig assemblage E isolate (P15) is publicly available (giardiadb.org [25]). For non-human patient isolates, an additional eight genome assemblies were derived from pools or single *G. duodenalis* cysts of assemblage C and D from dogs and one cat-derived assemblage A [12, 13]. Recently, a high-quality genomic reference for the distinct species *G. muris* has also been assembled [22]. The most complete and current reference genome is that of assemblage AI clone WB6/C6 (referred to as WB6 herein), which is a long-term laboratory cultured isolate. This reference captures an estimated 97 % of the total haploid genome size of WB6 and constitutes a near physical representation of its five chromosomes [21]. Equivalent high-coverage assembled genomic data for assemblage B parasites are lacking and the highly fragmented genomic assemblies of the long-term laboratory isolates GS and BAH15c1 [25] are the only publicly accessible references.

Difficulties in retrieving high-quality genome data for assemblage B parasites may in part result from allelic sequence heterozygosity (ASH), which poses a formidable challenge for assembly routines depending on sequencing technology. ASH in the assemblage B isolate GS reference genome is about 20-fold higher than in assemblage A based on the current reference genome of WB6 where ASH affects only 0.03 % of sites [8, 21]. The large differences in ASH between assemblages is corroborated by MLST data comparing assemblage A and B isolates (e.g. [26–29]).

Here, we addressed the lack of high-quality genome data sets for *G. duodenalis* by a hybrid PacBio/Illumina sequencing approach of four new assemblage A (one AI from a cat, and three AII from humans) and five new assemblage B isolates (from humans) derived by axenic culture from clinical samples. As result, we provide nine highly contiguous genome assemblies of new isolates of *G. duodenalis* assemblage A and B. In particular, we provide assembly of the most contiguous assemblage B genome currently available to be used for reference purposes.

## Methods

### Parasite culture, DNA preparation and sequencing

*G. duodenalis* trophozoites were grown in TYI-S-33 medium containing bovine serum as previously described [30]. Axenic cultures of the eight parasite isolates P344-B2, P387-C1, P392-H2, P424-A5, P427-B2, P458-E2, P064-F7 and P407-E2 were established from symptomatic and travel-related human giardiasis infections sampled between 2011 and 2015 by *in vitro* excystation and limiting dilution, but not formally cloned (see also below), as described previously [31, 32]. Patients had travelled to Nepal/India (isolate P387) or to the Dominican Republic (isolate P064), respectively; the travel history of the other patients is unknown. No further data on the isolates are available. These isolates were selected from a pool of preliminary genomes of 20 isolates because they represented different phylogenetic groups. In addition, one previously established isolate representing an assemblage AI-type (KO188) derived from a *G. duodenalis*-infected cat was included [33].

DNA was extracted from 5×10^8^ trophozoites using Qiagen Genomic-tip 20 G^−1^ columns according to the manufacturer’s recommendations. The quality of high-molecular-weight DNA was analysed on a 0.8 % agarose gel and DNA was quantified using a Quantus fluorometer (Promega).

For ‘long-read’ PacBio sequencing, 8 µg DNA was sent to GATC-Biotech (Konstanz, Germany) and sequencing was run on a PacBio RS II machine aiming at a minimum of 50-fold coverage of the estimated size of ~12 Mb of the haplotype genome. ‘Short-read’ Illumina sequencing of the patients’ isolates was performed at the sequencing facility MF2 of the Robert Koch-Institute on the same DNA preparations by using dual-indexed paired-end library construction using a Nextera Library Prep kit (Illumina). Average sample length of libraries was approximately 600–1000 bp and paired-end sequencing was done on an Illumina HiSeq 2000 instrument. Illumina sequencing of the KO-188 isolate was done by using a 150 bp paired-end technique, and sequencing was done on an Illumina HiSeq 4000 platform.

The dataset of Illumina reads of P344-B2, P387-C1, P392-H2, P424-A5, P427-B2 and P407-E2 was also supplemented with Illumina read data generated in an earlier project as described elsewhere [32].

### Reference sequences

The recent genome update of the assemblage AI isolate WB6 [21] (available on giardiadb.org [25]) and the genome sequences of the assemblage AII isolate DH and the assemblage B isolate GS [8] were used as reference sequences. The latter two sequence data sets were derived from giardiadb.org (release 46).

### Read demultiplexing and trimming

PacBio reads were provided as subread filtered data and used as provided. Short reads were quality-checked and trimmed with fastp (version 0.20.0 [34]).

### Assembly and polishing

PacBio subreads were aligned to each other with minimap2 (version 2.17-r941, parameter -x ava-ont [35]) and assembled with miniasm (version 0.3-r179 [36]). Assemblies were corrected with long and short reads: long reads were aligned to the assembled sequences with minimap2 (parameter -x map-pb) and assemblies were error-corrected once (P424, P458, KO188, P64, P392, P407) or three times (P387, P344, P427) with Racon (v1.4.3 [37]). Short reads were aligned with bowtie2 (version 2.3.5, parameter --very-sensitive, paired-end mode [38]) and assemblies were again error-corrected twice with either Racon (P387, P458, P344, P427; version 1.4.3 [37]) or Pilon (P424, KO188, P64, P392, P407;, version 1.23 [39]). We chose the different polishing rounds and algorithms based on the number of indels remaining after polishing and the number and average length of genes annotated. Assemblies of assemblage A and B were finally scaffolded separately with Ragout (version 2.2 [40]). The resulting scaffolds were ordered according to the WB reference sequence using Mauve (mauve_linux_snapshot_2015-02-13 [41]).

### Repeat identification

For each assembly, transposable elements (TEs) and repeat regions were identified with RepeatModeler (version 1.0.8 [42]). Candidate sequences from all assemblies were extracted and merged to identify TEs and repeats with RepeatMasker (version 4.0.6 [43]).

### Gene annotation

Assemblies were annotated with BRAKER (version 2.1.5 [44–46]) and comparative Augustus (version 3.3.3 [47]). Protein sequence data were used for both approaches. The protein sequences comprised the proteomes of *G. duodenalis* WB6 (giardiadb.org, release 46 [25]), P15 (Uniprot-ID: UP000008974), GScloneH7 (Uniprot-ID: UP000002488), DH (Uniprot-ID: UP000018320), GS (Uniprot-ID: UP000018040), BAH15c1 (Uniprot-ID: UP000070089), *G. muris* (Uniprot-ID: UP000315496) and the related diplomonad *Spironucleus salmonicida* (Uniprot-ID: UP000018208). For comparative Augustus, we trained Augustus with the annotation available for WB, DH and GSB (giardiaDB.org, release 46 [25]) and then followed previous instructions [47, 47]. Whole-genome alignments were done with progressive Cactus [48] using a phylogeny obtained with Co-Phylog [49]. Results from both annotations were joined with joingenes (part of BRAKER/Augustus) with the BRAKER annotation taking priority. Finally, genes were given consecutive IDs starting from GD_STRAINNAME_000010 and increasing by 10 at each gene (i.e. GD_STRAINNAME_000020 is the second gene). Proteins were annotated with PANNZER2 [50].

### Synteny

Synteny analysis was performed with MCScanX using default parameters (most recent binary retrieved in May 2019 [51]) by the pairwise determination of syntenic regions between isolates, which were inferred from the order of orthologues. Orthologues between all isolates were identified with DIAMOND reporting up to 100 alignments with a maximal e-value of 0.000001 (version 0.9.26, options -e 0.000001 k 100 --no-self-hits [52]). Only scaffolds larger than 100 kb are shown in the synteny plots.

### Orthology

Groups of orthologue genes were identified with OrthoMCL (version 2.0.9 [53]) as described in the software manual but performing the blast with DIAMOND with the same parameters used for the synteny.

### Estimation of dN/dS ratios

dN/dS ratios for groups of genes from either the orthology or the synteny analysis were calculated as described previously [54]. We excluded proteins smaller than 50 aa or proteins with a premature stop codon that reduced the protein to less than 90 % of its original size.

### Heterozygosity

To assess the heterozygosity of a given isolate, its short reads were aligned to its assembled genome with bowtie2 (version 2.3.5, parameter --very-sensitive, paired-end mode [38]). Duplicates were removed with Picard (version 1.140 [55]) and SNPs were called with FreeBayes setting the ploidy to 4, filtering for a minimal coverage of 10 and a minimal alternative allele frequency of 10 % (v1.3.2–40-gcce27fc, parameters --ploidy 4 --min-coverage 10 g 1000 --min-alternate-fraction 0.1 [56]). We set the cut-off for detecting alternative allele frequency/non-homozygosity comparatively low (8) to limit false negatives. This was based on the assumption that in a case in which one out of four alleles is different the true/expected allelic ratio is 25 % and further based on the expectation of achieving a coverage of 30. Assuming this, we used a binomial distribution model to estimate observed ratios that would still be compatible with an expected ratio of 25 %. In this example, the 95 % confidence interval spans values of observed ratio of about 9–42 %. Hence, a minimal alternative allele frequency of 10 % is a good indicator that a position is not homozygous.

### Molecular analysis

Genotyping of isolates was done using a common typing scheme based on the triosephosphate isomerase (*TPI),* beta-giardin (*BG*) and glutamate dehydrogenase (*GDH*) gene loci [57–59] using standard nested PCR techniques and subsequent bidirectional Sanger sequencing as described previously [29].

To confirm rearrangements in assemblage B genomes, we extracted the sequences spanning the breakpoint, aligned them with each other to create a consensus sequence and searched for this sequence in WB6. We then used the matched WB6 regions on chromosomes 1 and 5 and an additional 1.2 kb flanking region to design primers for a product spanning the breakpoint. Primers were designed with Geneious software tools (Biomatters). The following primer sequences were used: rearrangeB_1136 F, TTCAGCAGGGGACTATTCGC; and rearrangeB_5444 R, CGTTTATTGCGCGCCTACTC. PCR was done using 2.5 units of DreamTaq polymerase, 1× DreamTaq buffer (both Thermo Fisher), 0.2 mM dNTPs (Roth laboratories), 0.2 µM of each primer (Eurofins) and 100 pg DNA template. PCR was run using the following conditions: initial denaturation step of 5 min at 95 °C, followed by 35 cycles of denaturation for 1 min at 95 °C, annealing for 1 min at 59 °C and extension for 6 min at 72 °C. DNA was separated on a 0.8 % agarose gel and visualized using Midori green direct (Biozym).

### Data availability

Sequence data generated in this study were deposited at the NCBI Sequence Read Archive (SRA, https://www.ncbi.nlm.nih.gov/sra/PRJNA879307) and are accessible through the accession number PRJNA879307.

## Results

### Genome assemblies confirm high synteny and identify a major chromosomal translocation between chromosome 1 and 5 distinguishing assemblage A and B

Eight axenic isolates derived by limiting dilution from *G. duodenalis* patients’ stool samples and one isolate from a cat were subjected to genome sequencing using the platforms from Pacific Biosciences (PacBio) and Illumina. Coverage by PacBio long read-sequencing was 54- to 100-fold of the expected ~12 Mb genome size, and respective Illumina sequence coverage ranged between 140- and 550-fold (Table S1, available in the online version of this article).

**Fig. 1.**
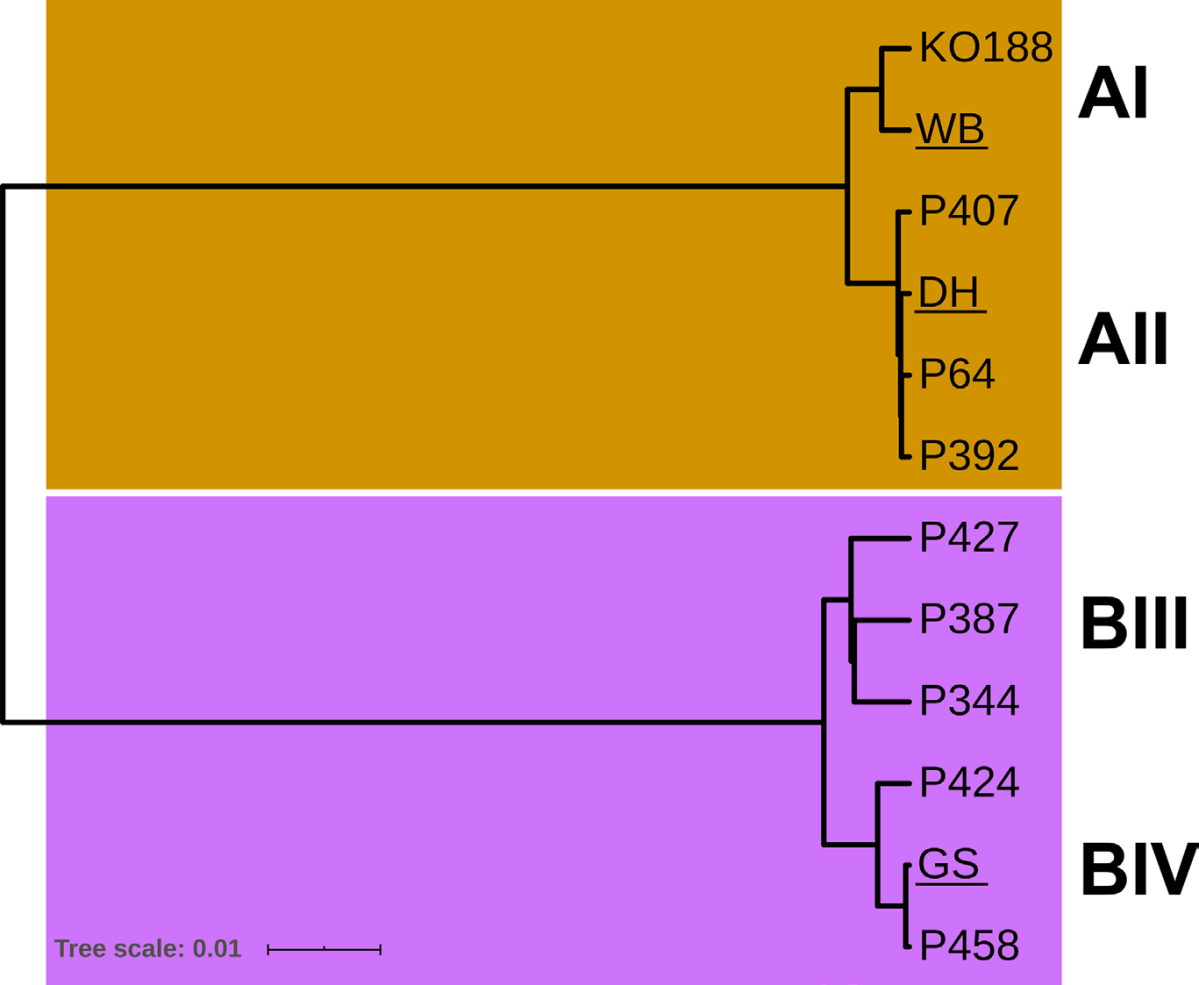
Whole genome-based phylogram of *G. duodenalis* isolates used in the present study. An assembly-free phylogenomic approach using Co-Phylog was applied to determine relatedness of the genomes of recent *G. duodenalis* isolates to reference genomes of isolates WB6 (assemblage AI), DH (assemblage AII) and GS (assemblage BIV). Tree scale represents nucleotide substitutions per site. Note that an alternative analysis using RAxML gave similar results (see Fig. S1).

Phylogenetic analysis using Co-Phylog [49], an assembly-free phylogenomic approach, and RAxML [60] confirmed that the parasite isolates were samplings of the major sub-assemblages causing disease in humans (Figs 1 and S1). Isolate KO188 belonged to assemblage AI and P64, P392 and P407 to AII. Isolates P424 and P458 clustered together with the sub-assemblage BIV group reference isolate GS, while P344, P387 and P427 belonged to sub-assemblage BIII.

**Fig. 2.**
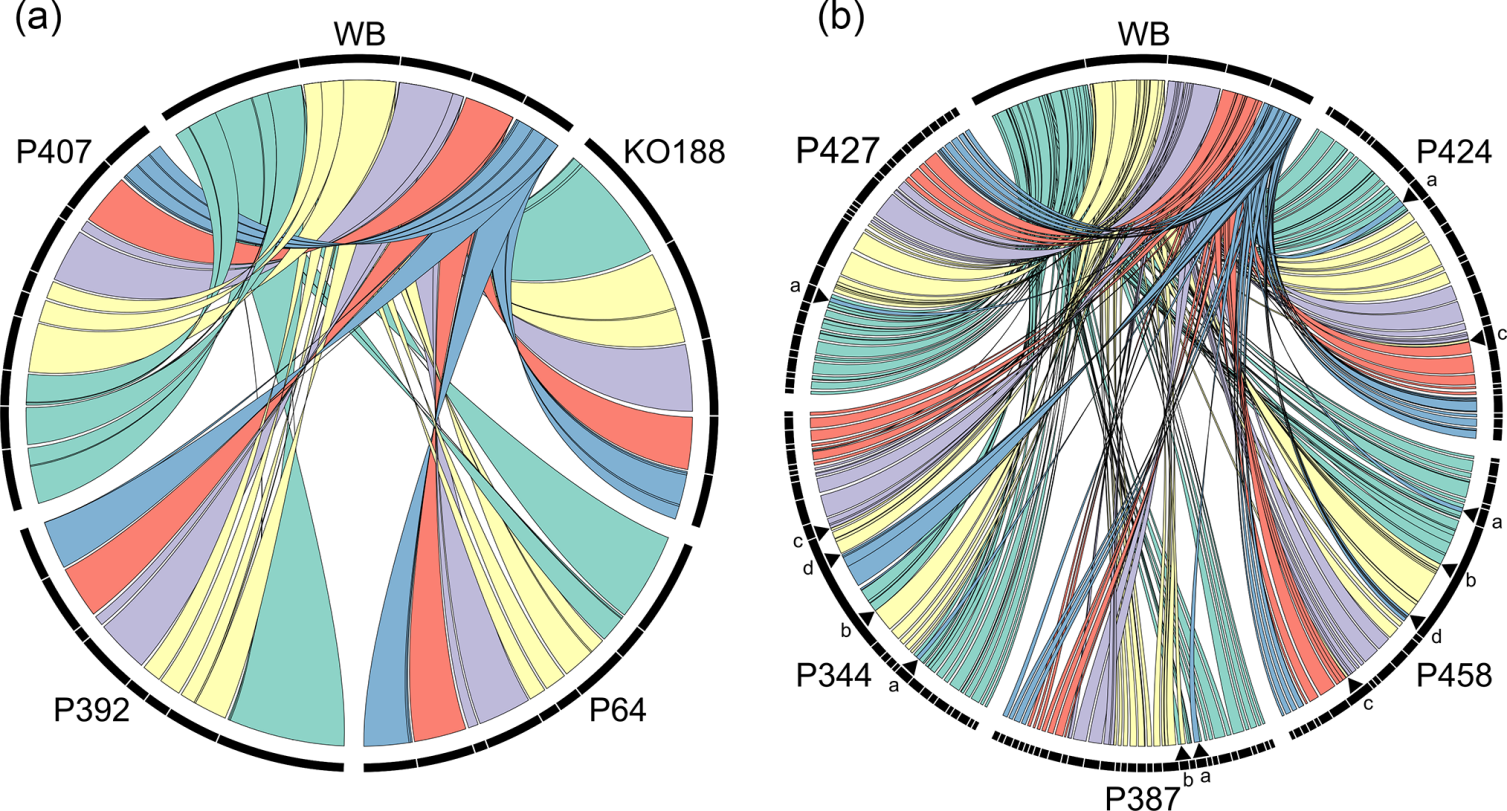
Circos plots illustrating syntenic regions of *G. duodenalis* isolates in comparison to reference isolate WB6. The syntenic regions of indicated assemblage A (**a**) and assemblage B (**b**) isolates to WB6 are presented in separate Circos plots. Note, for reference we used WB6 as a reference only, because no assemblage B genome is yet available at a similarly high-quality level. Similar rearrangements in assemblage B genomes compared to the assemblage A reference genome are highlighted by arrowheads and lower-case letters a–d. Rearrangement ‘a’ was present in all assemblage B genomes and was experimentally validated by PCR (see Fig. 3). Other rearrangements were not present in all assemblage B genomes and were therefore not further investigated.

*De novo* assembly of error-corrected PacBio data followed by separate scaffolding for assemblage A and B resulted in assembled genomes that ranged from 10.8 to 13.7 Mb in size and were fragmented over 14–132 scaffolds. Table 1 summarizes the characteristics in comparison to public *G. duodenalis* genome data available at Giardiadb.org [25]. Synteny to the reference genome for assemblage AI (isolate WB6 recently improved to a near physical representation of the five chromosomes [21]) was analysed next. The analysis was based on predicted gene product orthologue order since direct sequence comparison between assemblages was precluded by the respective low average nucleotide identities (<80 %, as also previously noted for WB6 and GS by others [10]). For assemblage AI isolate KO188, and AII isolates P64, P392 and P407, the overall congruence with the reference WB6 genome was 93–95% and reads represented 94–99 % of their own respective genome assemblies (Fig. 2a, Tables 1 and S2). For KO188, the five largest scaffolds corresponded closely to the five chromosome equivalent scaffolds of the WB6 reference.

**Fig. 3.**
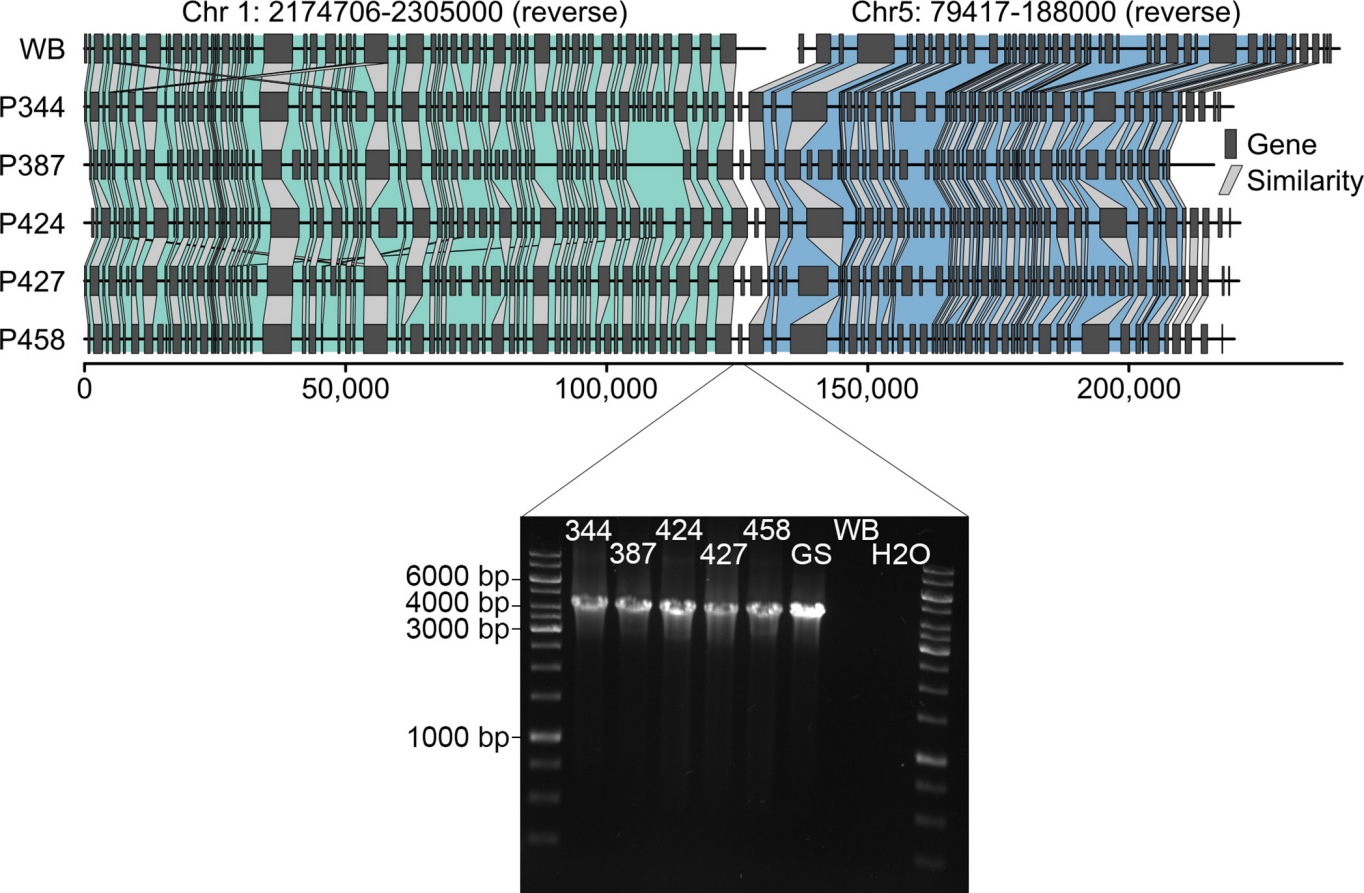
Representation and verification of a major genome rearrangement in assemblage B isolates compared to the WB6 reference. The genome rearrangement in chromosome 5 of all assemblage B isolates (referred to as rearrangement ‘a’ in Fig. 2b) was verified by PCR, amplifying a 4309 bp long fragment spanning the overlapping region of chromosomes 1 and 5 of the respective WB6 genome. In addition to the assemblage B genomes generated in this work, DNA of assemblage B lab strain GS was included in the PCR for further validation. The amplified region spans over three coding regions for unknown proteins.

**Table 1.** Description of genome assemblies

Isolates	Sub-assemblage	No. of contigs	Contig N50	Contig N90	Assembly size (bp)	No. of scaffolds	Scaffold N50	Scaffold N90	No. of gaps in scaffolds	Total gap size in scaffold	No. of coding genes	Gene size (aa)	Coding density (%)
**Assemblage A**													
KO188	AI	30	755665	349 063	11746015	14	2815069	1452157	16	34 781	5151	615.7	80.0
P64	AII	54	449560	149808	11412007	28	1565103	518187	27	96579	5162	599.1	81.3
P392	AII	49	769973	239635	12012982	37	1600849	596336	14	39 713	5465	581.1	79.3
P407	AII	40	888328	178532	11744889	28	1462488	552 701	12	18 079	5336	593.2	80.9
**Assemblage B**													
P344	BIII	141	542592	66183	13756513	111	448428	43394	64	327130	6096	542.5	72.1
P387	BIII	179	203840	65799	10807475	124	174582	45106	98	634969	4947	538.6	74.0
P424	BIV	56	621392	200 270	12730877	63	563076	123334	11	6625	5489	592.1	76.6
P427	BIII	166	55835	108487	12751136	132	328615	43198	78	455107	5747	543.9	73.5
P458	BIV	98	208 205	324 946	12 803 159	80	650 350	78 399	44	82 076	6116	551.6	79.1
References*													
WB	AI	38	2761001	1 485 438	12 078186	35					4965	635.1	78.3
DH	AII	239	117284	30 360	10703894						5147	591.1	85.3
GS	BIV	543	58544	8867	12009633						6098	540.0	82.3

*The following resources for references were used: WB6 [21], DH and GS [8]. The latter two sequence data sets were derived from giardiadb.org (release 46).

Plotting the assembled genomes of the assemblage B isolates in relation to the WB6 reference enabled visualization of the overall high synteny also between assemblages (Fig. 2b). Depending on the isolate, syntenic blocks corresponded to 79–93 % of the WB6 genome but represented only 81–88 % of their own assemblies (Table S2). The comparably low fragmentation of our assemblage B assemblies revealed a major inter-chromosomal rearrangement as a characteristic and consistent difference to the assemblage AI WB6 reference (translocation ‘a’ in Fig. 2b). We verified this altered arrangement at chromosomal DNA level by PCR (Fig. 3).

We next investigated the distribution of DNA repeats over the assemblies. Patterns were distinct between isolates but showed notable similarities at (sub)-assemblage level (Fig. S2). Thus, average nucleotide identities, major chromosomal rearrangements and repeated DNA patterns are distinct characteristics that distinguish assemblage A and B genomes.

### Allelic sequence heterozygosity analysis identifies P424 as the first example of an assemblage B-type isolate with extremely low heterozygosity

ASH has been reported to differ quantitatively between assemblages [8, 21]. We therefore analysed ASH in our genome data sets. Variant bases were called when coverage was >10-fold at a sequence position and variant bases were present in >10 % of respective reads (Table 2). As *Giardia* is tetraploid, variant bases may exist in a one-to-one or one-to-three ratio; thus, both ratios were considered. All assemblage A isolates exhibited very low ASH affecting less than 0.007 % of positions in three of the four isolates and 0.074 % in isolate P392. Consistent with our expectations, ASH values were mostly much higher in assemblage B genomes ranging from 0.259 % in assemblage BIV isolate P458 to values above 1.5 % in assemblage BIII isolates P344, P427 and P387. Surprisingly, the genome sequence of isolate P424, also belonging to assemblage BIV, displayed only 232 variant positions in a total sequence length of 12 138 704 bp, reducing its ASH value to 0.002 % (Table 2).

**Table 2.** Allelic sequence heterozygosity (ASH) in *G. duodenalis* genome datasets

		No. of variable sites*				No. covered (bp)		ASH* (%)					Distance between sites (no. of sites)		
Isolate	Sub-assemblage	1 : 1	1 : 3	> one GT	Total	Illumina	Pacbio	1 : 1	1 : 3	> one GT	Total	Coding	< 300 bp	300-10 kb	>10 kb
**Assemblage A**															
KO188	AI	128	346	14	488	11 400 775	10 882 370	0.001	0.003	0	0.004	46.10	381	38	64
P64	AII	398	333	16	747	10 919 399	10 321 636	0.004	0.003	0	0.007	38.46	511	79	142
P407	AII	139	159	7	305	11 255 959	10 786 915	0.001	0.001	0	0.003	41.32	215	29	49
P392	AII	3887	4421	127	8435	11 353 893	10 832 231	0.034	0.039	0.001	0.074	76.74	6588	1655	172
**Assemblage B**															
P344	BIII	84 340	104 500	3961	192 801	12 647 461	11 751 273	0.667	0.826	0.031	1.524	84.06	189 332	3298	41
P387	BIII	74 218	102 521	4487	181 226	9 962 759	8 456 530	0.745	1.029	0.045	1.819	81.97	178 346	2710	24
P424	BIV	111	104	17	232	12 138 704	11 166 760	0.001	0.001	0	0.002	18.61	132	27	41
P427	BIII	75 569	92 121	3476	171 166	11 840 257	10 688 359	0.638	0.778	0.029	1.446	84.11	167 397	3579	42
P458	BIV	13 475	18 065	104	31 644	12 219 296	11 129 574	0.11	0.148	0.001	0.259	81.79	26 710	4685	170

*As *Giardia* is tetraploid, variant bases may exist in a one-to-one or one-to-three ratio and thus both ratios were considered [‘> one GT’ indicates more than one genotype (GT)].

We next used PCR for independent confirmation of the extremely low ASH observed in isolate P424 and the extremely high number of variant positions in isolates P344, P387 and P427. To this end, all isolates were subjected to MLST analysis using *TPI*, *GDH* and *BG* typing PCRs. As shown in Fig. S3, no ambiguous positions were found in the total of 1358 bp covered by this approach for P424 while the other isolates showed between three and nine polymorphic positions, i.e. 0.2–0.6 %.

**Fig. 4.**
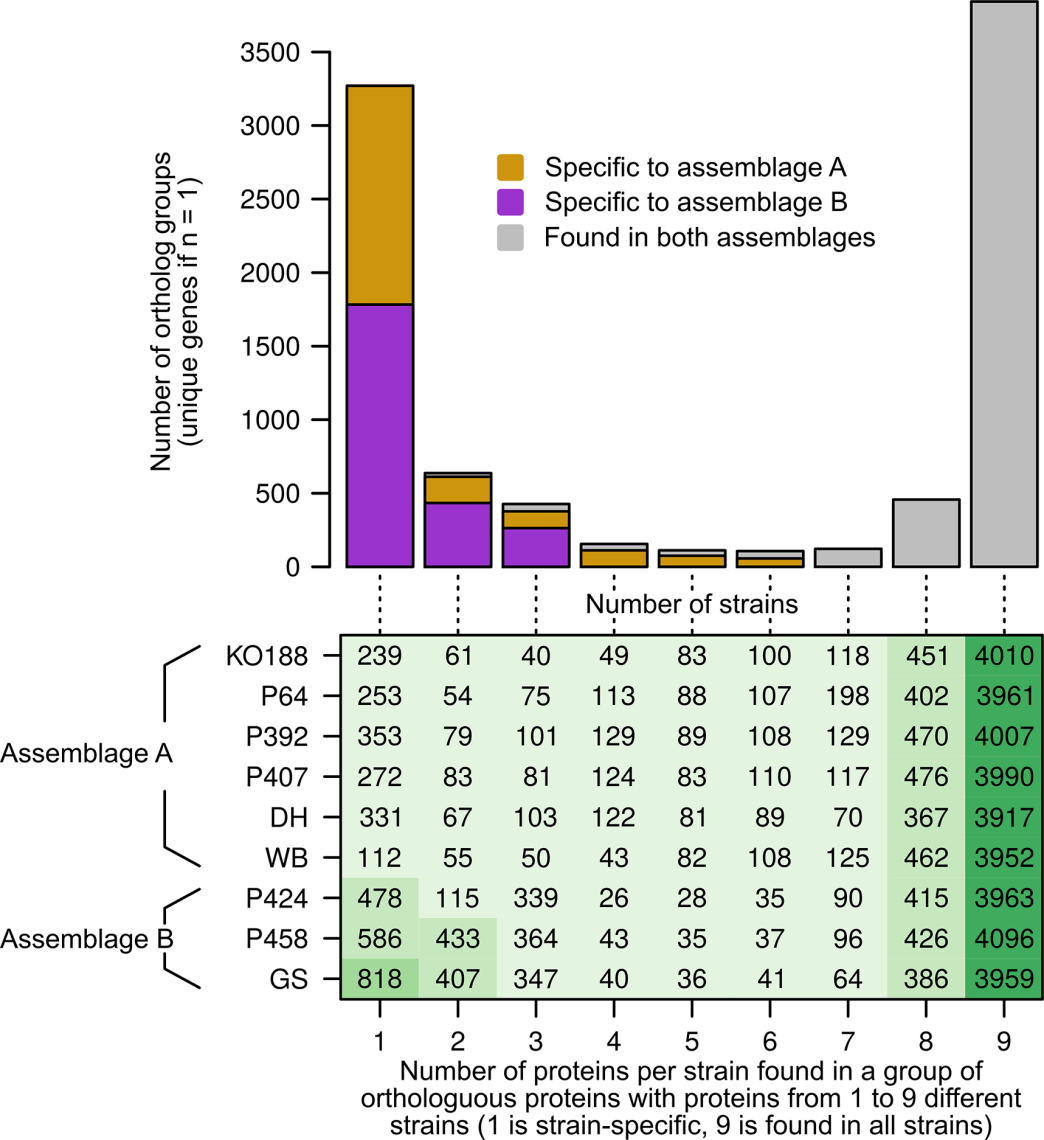
Number of orthologous proteins per isolate reveals representation of pan- and assemblage-specific accessory genes. Due to low average nucleotide identity between assemblage A and B, the analysis was based on the predicted gene product orthologues (Table S3). Numbers of genes found in orthologue groups per isolate were compared to all other isolates in a stepwise manner and results were plotted in the table (lower panel). We further assessed if numbers of orthologous groups [one orthologous group may comprise one or more protein; if a gene is unique (*n*=1) the orthologous group comprises one gene] are only found in assemblage A (orange), B (pink) or both assemblages (grey) and results are presented in the bar graph (upper panel). Note, as numbers of proteins per isolate may differ depending on the orthologous group, protein numbers per isolate differ in row 9 representing proteins from all orthologous groups found in all isolates (core=3845 orthologous groups). For instance, isolate KO188 comprises 4010 proteins (paralogues) within 3845 orthologous groups found in all nine isolates. Due to uncertainties as to whether isolates P344, P387 and P427 represent clonal populations, the data from these isolates were removed from the dataset shown. Refer to Fig. S4 for the full analysis including data from these isolates.

ASH sites have been reported to be non-randomly distributed [9]. Here, the distribution and partitioning over coding and non-coding sequence differed significantly between genomes. Polymorphic sites tended to be concentrated in non-coding sequence in assemblage A genomes with values ranging from 54 to 62 % (Table 2). The exception was isolate P392 in which 77 % of variant bases were detected in coding sequences (Table 2). In contrast, ASH occurrence was most consistent in assemblage B genomes (P344, P387, P427 and P458; Fig. 4b; Table 2) with a random distribution with P424 being the exception.

### Annotation and derivation of *G. duodenalis* assemblage-level pan, core and accessory protein coding genomes

The new genomes were annotated based on protein sequence comparisons to predicted annotated proteomes of WB6 and other sequenced *G. duodenalis* isolates, of *G. muris* and of *S. salmonicida*. This reference-based automated annotation assigned between 4947 (P387) and 6096 (P344) genes depending on the isolate genome data set (Table 1), which is a range congruent with gene counts in published *Giardia* genomes [8, 11, 61] reported at 4965 and 6098 genes for WB6 and GS, respectively (Table 1).

Next, orthologues and paralogues were identified by OrthoMCL (Table S3). Orthologue gene groups were classified into groups with members in all isolates. Such orthologous gene groups operationally defined the core genome and the respective accessory genome of gene groups and singletons exhibiting presence–absence variation between isolates (Fig. 4; see also Table S3 for respective predicted orthologue gene groups and genes).

The core genome common to assemblage A and B genomes was represented by 3017 orthologue groups (Fig. S4; Table S3). The respective gene sets that included paralogues encoded between 3077 and 3370 proteins (Fig. S4). Presence–absence variation was predicted for 3353 orthologue gene groups that consisted of at least two ortho- or paralogous sequences, and for 4953 genes assigned as singletons to only one of the genomes (Fig. S4 and Table S3). There were 98 orthologue groups that were consistently identified either only in A (52 orthologue groups) or only in B (46 orthologue groups) genomes (Table S3). Orthologue groups assigned only to (sub)-assemblages totalled 38 in AI (WB6 reference and KO188 genomes only), 74 in AII (DH reference, P64, P392 and P407), 32 in BIII (P344, P387 and P427) and 117 in BIV (GS reference, P424 and P458).

Due to the high average ASH number in assemblage BIII genomes, we considered the presence of intra-assemblage mixed infections despite the establishment of the cultures by limiting dilution. Indeed, cloning and sequencing of the two nitroreductase genes revealed an allelic sequence number >4 for the assemblage BIII isolates, but not the assemblage BIV isolates investigated in the present project [62]. We re-analysed the orthologue groups omitting the assemblage BIII isolates P387, P344 and P427 in order to avoid false estimation of group-specific genes in assemblage B (Fig. 4). While the overall results remained similar, the number of core genes increased and the number of specific genes decreased as expected. The core genome now represented 3845 orthologue groups, and the respective gene sets comprised between 3917 to 4096 predicted proteins (Fig. 4; Table S3). Predicted presence–absence variation with two or more orthologues consisted of 3148 orthologue groups and 2965 were assigned as ‘unique’ to only one of the remaining genomes (Fig. 4; Table S3). The number of orthologue groups consistently found only in A (57) or B (263) assemblage genomes was increased to 320. The number of orthologue groups assigned only to (sub)-assemblages changed slightly to 38 in AI (WB and KO188), to 79 in AII (DH, P64, P392 and P407) and to 263 in BIV (GS, P424 and P458).

**Fig. 5.**
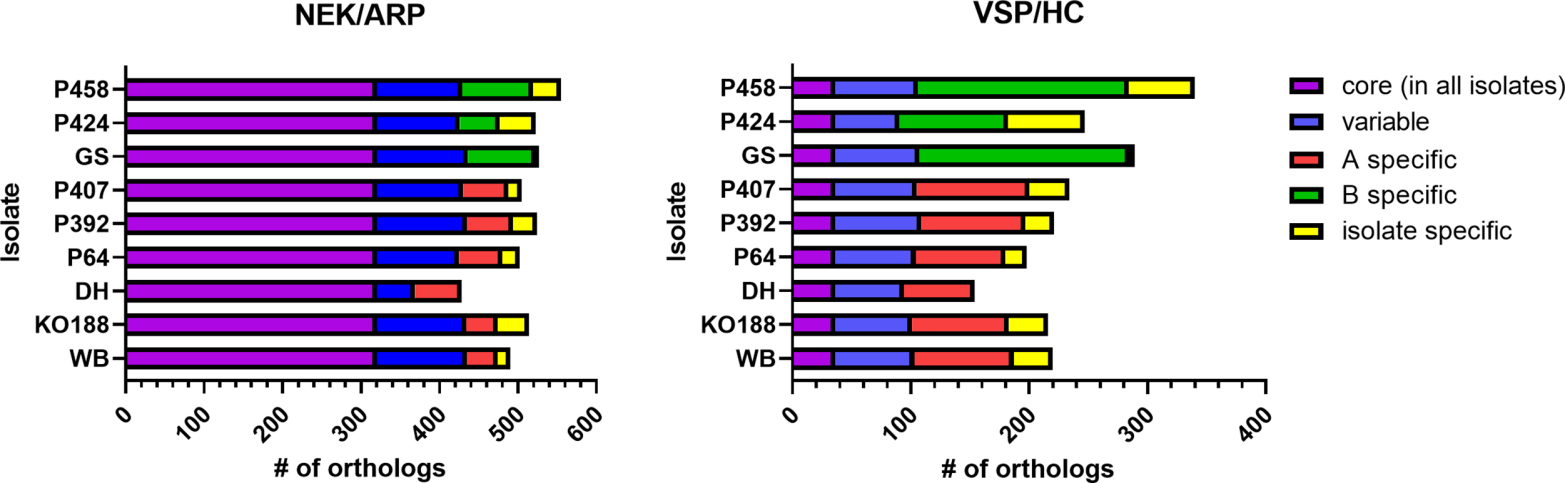
Number of orthologue groups of known major *Giardia* protein families by isolate. Members of the major protein families NEK kinases and ankyrin repeat proteins (NEK/ARP) and variant surface proteins and high cysteine proteins (VSP/HC) are presented dependent on their representation in core, variable, assemblage or isolate-specific groups. Due to uncertainties as to whether isolates P344, P387 and P427 represent clonal populations, the data from these isolates were removed from the dataset shown. Refer to Fig. S5 for the full analysis including data from these isolates.

Orthologues of large gene families in *Giardia*, such as the variant surface protein (VSP) family, high cysteine (membrane) proteins (HC), ankyrin repeat domain-containing protein (ARP) family, and the NEK kinase protein (NEK) family dominated the annotations of these differential gene sets (Figs 5 and S4; Table S3). In Fig. 5 the data are plotted without the BIII isolates and revealed 154–340 VSP/HC orthologues and 428–555 NEK/ARP orthologue groups. We noted that a total number of 320 NEK/ARP (58 –75 % of all NEK/ARP) orthologue groups belonged to the core genome content whereas only a total number of 36 VSP/HC (11 –23 % of all VSP/HC) were shared orthologues (Fig. 5; Table S3). Reversely, the proportion of assemblage A-specific (11 –17 %) and assemblage B-specific (17 –23 %) NEK/ARP orthologues was lower than assemblage-specific proportions of VSP/HC orthologues which comprised 39–55 % (assemblage A-specific) and 63–69 % (assemblage B-specific). Fig. S5 gives a plot of the complete data sets, including assemblage BIII isolates.

Hypothetical, or non-homologous, proteins were another prominent class of the orthologue groups in the assemblage/sub-assemblage-specific genes. In assemblage A genomes, nine of 57 assemblage A-specific orthologue groups are found in all assemblage A isolates (or eight of 52 when including the BIII genome datasets). In (sub)-assemblage AI and AII genomes they numbered 15 of 38 (15 of 38) and 33 of 79 (31 of 74), respectively. The fraction of orthologues of assemblage B (in the dataset excluding BIII genomes) that showed no homology to other genes was 45 of 263 [or nine of 46 when including the BIII genome datasets; the numbers for (sub)-assemblage BIII and BIV genomes comprised 10 of 31 and 22 of 117, respectively].

Besides members of these large gene families, orthologues assigned known specific functions such as enzymatic activities present in one but not the other assemblage were also observed. Genes encoding arginases, 2,5-diketo-d-gluconic acid reductases and carboxymuconolactone decarboxylase and lactonases have been reported to differentiate assemblage B from A genomes [22]. Here, arginase was confirmed to only be predicted for assemblage B genomes. Genes annotated as homoserine lactonases, as carboxymuconolactone decarboxylases and genes assigned a 2,5-diketo-d-gluconic acid or non-family 1 aldo-/keto reductase function were present in assemblage B genomes but absent in assemblage A genomes.

Taken together, genome annotation in the newly assembled *Giardia* genomes confirmed the representation of large gene families in *Giardia* assemblage A and B and revealed core and assemblage/isolate-specific orthologue proteins that will help to decipher biological differences.

## Discussion

We report highly contiguous genomic sequences and respective assemblies for nine *G. duodenalis* isolates, including eight clinical human samples and one cat sample, which addresses a significant knowledge gap. The new genomes represent a sampling of the major human-pathogenic parasite (sub-)assemblages AI, AII and B as shown by assembly-free phylogenetic analyses. Predicted size, gene content and general features of the genomes corroborate currently available reference genome data. High-quality assemblage B genomes have been a resource much desired in this field. Our genome assemblies show low fragmentation comparable to the most advanced assemblage AI-type reference genome of isolate WB6.

It is of debate whether *G. duodenalis* is composed of eight genetically distinct assemblages or whether the term ‘assemblage’ is a misnomer for a collection of separate species historically defined based on host range [1, 4]. The first draft of the assemblage B isolate GS genome published in 2009 [10] with its phylogenomic interpretation of low average nucleotide identity first supported the concept of different species for assemblage A and B. This was corroborated by recent re-analysis of further assemblage B genome data [61]. Our new genomic data add a consistent chromosome-level translocation and distinct genome distribution of repeats as further phylogenetic signals [63, 64] to support this view. However, the phylogenetic approach is only one of more than 20 different approaches for the definition of species [65, 66] and is not conclusive with respect to their taxonomic delimitation.

To the best of our knowledge, the assembly of isolate P424 represents the first assemblage B near haplotype genome sequence. The observation that this genome has extremely low ASH in comparison to other available assemblage B isolate genome sequences challenges the current notion that a high ASH level is a distinctive characteristic of assemblage B. Of note, P424 is not a highly singular example but, based on recent data, belongs to a subpopulation of BIV isolates exhibiting significantly fewer ASH positions detectable by MLST [29]. This highlights that still much is to be learnt to understand the parasites’ population genomic landscape and its relationship to taxonomic classification.

We used a hybrid approach combining long-read sequences generated by PacBio technology and short-read sequences by Illumina to build high-quality genome assemblies. A similar approach was recently applied to three different *Giardia* isolates combining long-read sequences from MinIon instead of PacBio [15]. The authors highlighted that each of the re-sequenced reference isolates WB and GS and one newly sequenced assemblage AI isolate were ‘shown to contain structural variant regions enriched for variant-specific surface protein’ genes [15]. Our approach delivers consensus assemblies that are averaged representations of respective, possibly individually variant genomes. While the former approach reveals the genomic plasticity of *Giardia*, the latter appears more informative for the definition of gene content, as well as the pangenome, and core and accessory genomes.

Comparative analyses of the gene content of our new isolates to the reference genomes of WB6, DH and GS revealed that 53–65 % of genes encoded proteins with corresponding orthologues in all isolates. In contrast, the presence–absence variation segregated the A and B assemblages as well as the accessory genomes of (sub)-assemblages. As a whole, our data significantly extend the understanding of the core genome of the genus *Giardia* and suggest distinguishable (tentative species-specific) pan genomes.

Functional annotation is required for the interpretation and discussion of biological consequences of these genomic differences in gene content and presence–absence variation. Here, functional assignments refer to the respective annotations of the currently best annotated genome (i.e. that of WB6). However, general limitations of automated annotation approaches apply. Automated genome annotation though common practice has been highlighted as ‘*A blessing and a curse*’ [67]. Such automated annotations are a curse in particular in the functional annotation of paralogues. Estimates for incorrect prediction of function reach up to 25%, and the propagation of such faithful but false calls into higher order databases cannot be prevented. Our functional inferences should therefore be read bearing this caveat in mind.

One major limitation of our study is that, against expectations, some of the cultured assemblage B isolates apparently do not represent clonal cell lines. Culture efficiency of *Giardia* parasites is generally very low and may be prone to culture bias. As of today only assemblage A and B isolates and assemblage E can be successfully cultured axenically [68]. Typically, new *Giardia* isolate cultures are established by *in vitro* excystation and cultured as bulk populations (described in the very first publications), or isolated by cyst infection in rodents with recovery of trophozoites from animal small intestine [30, 69, 70]. Clonal populations are not generated regularly. The cultures of the current study were generated by limiting dilution of excysted trophozoites to both avoid cross-contamination and to fractionate the isolates. However, the isolates were not formally clonal populations. In the case of assemblages AI, AII and BIV this approach generated quasi-clonal populations, as suggested by the low ASH number and acceptable *de novo* assembly efficiency. However, for the selected assemblage BIII isolates, the recent analysis of the two nitroreductase genes by cloning of PCR products from the said isolates’ genomic DNA revealed more than four alleles. This indicates derivation of the lines from a possibly underlying mixed infection [62]. The latter observation could also be explained by various other explanations, including possible gene duplication that may have hampered sequence analyses. This should be kept in mind while interpreting the generated data. We recently estimated the likelihood of intra-assemblage B mixed infections in patients at roughly 50 % [29]. Therefore, not all our BIII lines may be derived from true mixed infections. An alternative explanation for our findings could be that the high ASH may be the result of a higher recombinational/mutational rate during culture of these specific isolates. Not much is known about DNA repair mechanisms and quality control steps during DNA replication in *Giardia*, especially within assemblage B isolates. It should be noted that one early study using the assemblage AI (WB6) genome revealed that the minimal kinome of *Giardia* lost important kinases in comparison to other excavata, including ‘kinases involved in central biological functions, such as DNA repair, transcription, splicing, and mitochondrial metabolism’ [71]. It is therefore possible that the ‘mixed’ phenotype emanated due to insufficient DNA repair mechanisms. Alternatively, ASH content may also reflect times elapsed after genetic homogenization as asexual replication in polyploid organisms will accumulate ASH over time [72]. Different observations show that *Giardia* assemblage A parasites exchange genetic material between nuclei and may perform some sort of parasexual reproduction (diplomixis) [73]. Inter-nuclear genetic exchange may partly explain the ‘homozygote’ nature of assemblage A parasites, as seen in recent multi-locus sequencing analyses [5, 29]. For assemblage B, it is unknown whether or to what extent the exchange of genetic material occurs between nuclei or isolates. Also, different assemblage B isolates may vary in their ability to exchange genetic material. Future studies using clonal populations derived from the described A and B isolates will possibly help to resolve these questions.

We chose to include potentially non-clonal genomes for two reasons. First, the approaches used here to culture and analyse genomes from *Giardia* clinical isolates are standard and commonplace in the field. Second, we aim to draw attention to potential non-clonal isolates confounding forthcoming or existing *Giardia* genome datasets. The analytical approaches used here can aid in determining clonality in sequenced isolates. The ‘mixed’ genomes assembled well and are informative in aspects that are common features of assemblage B parasites, such as the apparent chromosomal rearrangement distinguishing assemblage A and B (Figs 2 and 3). For the subsequent gene annotation analysis, however, we analysed the data with and without said genomes as described in the Results to avoid any false interpretation.

The finding that assemblage A and B segregated based on presence–absence variation in genes predicted to encode metabolic enzymes such as arginase, or aldo-/ketolases complements observations reported recently based on comparative analysis of the *G. muris* genome [22]. It is also evident that the large NEK/ARP and VSP/HC gene families in *Giardia* show separation between the two assemblage types, thereby supporting earlier studies [71]. For example, approximately 50 % of the NEK/ARP kinase family are in the ‘core’ genome identified in the present study. The remaining NEK/ARP kinases are either distributed over separate isolates or are assemblage/isolate-specific. It has been reported that the NEK protein family shows largely diverse sequence homologies and that most of the proteins have lost their catalytic function [71]. The NEK protein family has largely expanded in *Giardia* (as in other flagellates) compared to other eukaryotes (e.g. *Saccharomyces cerevisiae* and *Homo sapiens* which possess only one and 11 NEK proteins, respectively). NEK proteins are associated with functional roles in mitosis and flagella formation [71, 74]. VSP/HC proteins represent only a minor part of the core genomes and are largely assemblage-specific. VSP expression and surface exposure is a regulated process in *Giardia* and is thought to mainly act as part of immune escape mechanisms and cell surface protection [75]. The specific roles the assemblage-specific NEK and VSP proteins play in the different assemblages remain to be investigated. The functional roles of the many NEKs predicted to lack catalytic function are of particular interest.

In summary, hybrid sequencing data from long-read sequences by PacBio and short-read sequencing by Illumina technology allowed the generation of nine highly contiguous assemblies of genomes of tetraploid *G. duodenalis* isolates that represent a sampling of the main human pathogenic assemblage types AI, AII and B. Overall structure and gene content confirms and significantly complements data from available genomes. The data contribute to the definition of pan, core and accessory genomes. Notably, the assemblage B isolate P424 sequence data represent a desired near-haplotype genome for this assemblage.

## Supplementary Data

Supplementary material 1

Supplementary material 2
