# Supplementary material for: Highly contiguous genomes of human clinical isolates of Giardia duodenalis reveal assemblage- and sub-assemblage-specific presence–absence variation in protein-coding genes
Source: Microb Genom. 2023 Mar 28;9(3):mgen000963. doi: 10.1099/mgen.0.000963 (PMC10132058; doi:10.1099/mgen.0.000963)
Supplement: Supplementary material 1 [file mgen-9-963-s001.pdf]

Supplementary information

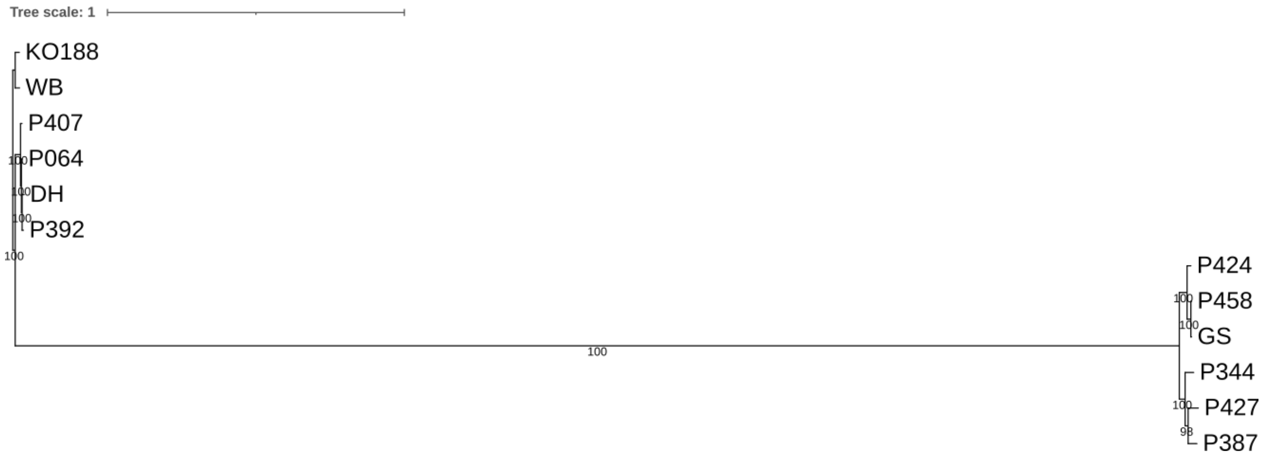

Figure S1: Maximum likelihood based phylogram of *G. duodenalis* isolates used in the present study and corresponding reference sequences (WB: NCBI GCA_000002435.2 (assemblage AI); DH: NCBI ASM49871v1 GCA_000498715.1 (assemblage AII); GS: NCBI ASM49873v1 GCA_000498735.1 (assemblage BIV)). The tree was inferred using sequence information that were available across all analyzed samples and built with RAxML (version 8.2.12 [1]).

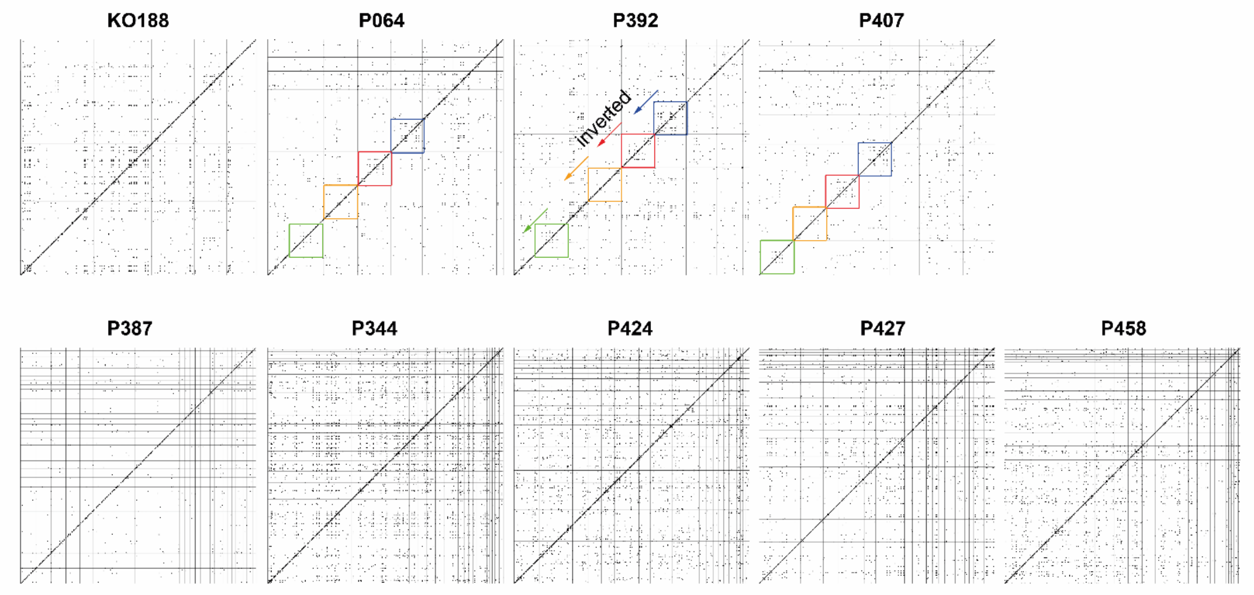

Fig. S2. Plots of repeat sequences by isolate. Assemblies were used to identify repeat sequences using RepeatModeler and RepeatMasker as described in the methods. Upper row shows Assemblage A isolates and lower row assemblage B isolates. Note pattern similarities in sub-assemblage AII isolates P064, P392 and P407 as indicated by colored squares. Arrows indicate that patterns are inverted in P392.

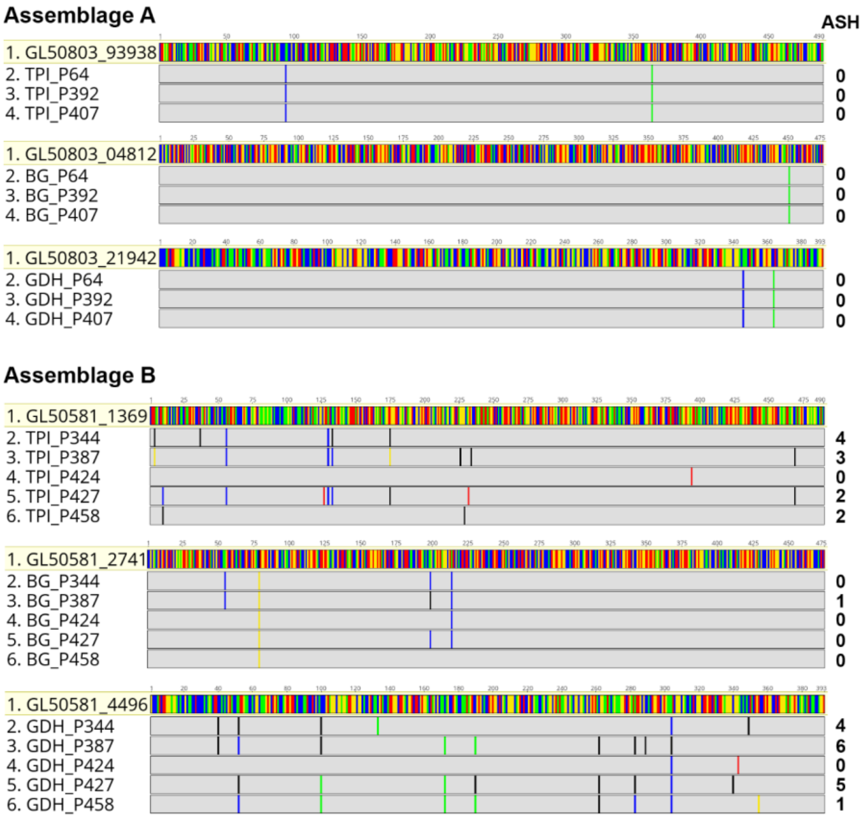

Fig. S3: Genotyping of *G. duodenalis* isolates at three common typing loci confirms very low ASH in assemblage B isolate P424. Genotyping at TPI, BG and GDH gene loci were performed by nested PCR and bidirectional Sanger sequencing. Chromatograms were individually analyzed and nucleotide variation to the presented references genes of assemblage A (isolate WB) and assemblage B (isolate GS) are marked for A (red), T (green), C (blue), G (yellow) and for ambiguous nucleotides (black). Following ambiguous nucleotides (IUPAC code) were noted (position based on PCR product without primer sequence): TPI_P344 (R at nucleotide position 4 and 175, Y at position 37 and 133), TPI_P387 (R at 226 and 234, Y at 469), TPI_P427 (R at 175, Y at 469), TPI_P458 (Y at 10, R at 229), BG_P387 (Y at 199), GDH_P344 (Y at 40, 52, 100, 349), GDH_P387 (Y at 40, 100, 262, 283, 289, 304), GDH_P427 (Y at 52, 190, 262, 283, 340), GDH_P458 (Y at 262).

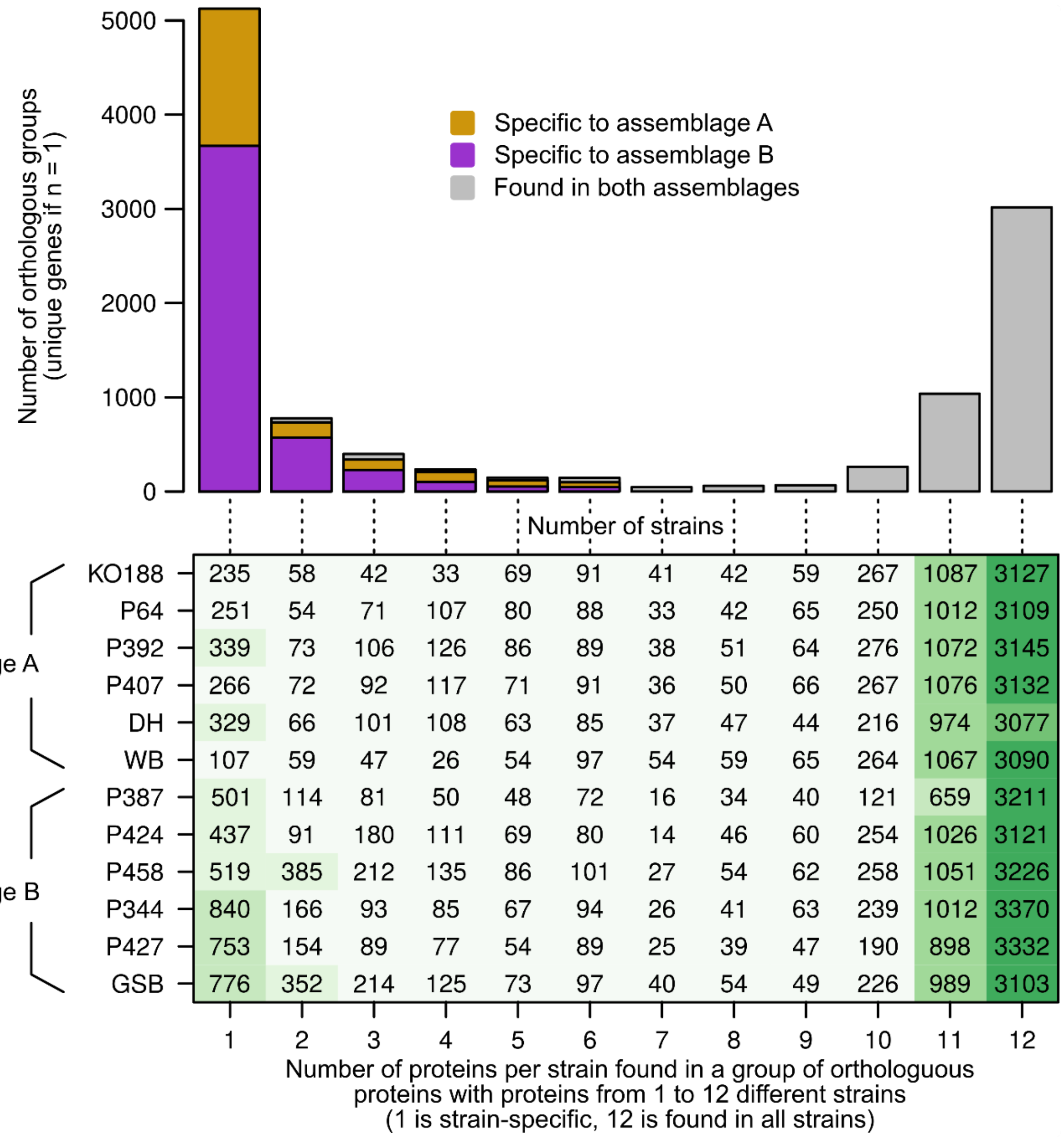

Assemblage	Strain	1	2	3	4	5	6	7	8	9	10	11	12
Assemblage A	KO188	235	58	42	33	69	91	41	42	59	267	1087	3127
	P64	251	54	71	107	80	88	33	42	65	250	1012	3109
	P392	339	73	106	126	86	89	38	51	64	276	1072	3145
	P407	266	72	92	117	71	91	36	50	66	267	1076	3132
	DH	329	66	101	108	63	85	37	47	44	216	974	3077
	WB	107	59	47	26	54	97	54	59	65	264	1067	3090
Assemblage B	P387	501	114	81	50	48	72	16	34	40	121	659	3211
	P424	437	91	180	111	69	80	14	46	60	254	1026	3121
	P458	519	385	212	135	86	101	27	54	62	258	1051	3226
	P344	840	166	93	85	67	94	26	41	63	239	1012	3370
	P427	753	154	89	77	54	89	25	39	47	190	898	3332
	GSB	776	352	214	125	73	97	40	54	49	226	989	3103

Number of proteins per strain found in a group of orthologuous proteins with proteins from 1 to 12 different strains (1 is strain-specific, 12 is found in all strains)

Fig. S4. Number of orthologous proteins per isolate reveals representation of pan- and assemblage specific accessory genes. Due to low average nucleotide identity between assemblage A and B the analysis was based on the predicted gene product orthologs (ref. Supplementary table 3). Number of genes found in ortholog groups per isolate were compared to all other isolates in stepwise manner and results were plotted in the table (lower panel). It was further assessed if numbers of orthologous groups (one orthologous group may comprise one or more protein, if gene is unique (n=1) the orthologous group comprises one gene) are only found in assemblage A (orange), B (pink) or both assemblages (grey) and results were presented in the bar graph (upper panel). Note, as numbers of proteins per isolate may differ depending on the orthologous group, thus protein numbers per isolate differ in row 9 representing proteins from all orthologous groups found in all isolates (core = 3017 orthologous groups). For instance, isolate KO188 comprises 3127 proteins (paralogs) within 3017 orthologous groups found in all 9 isolates.

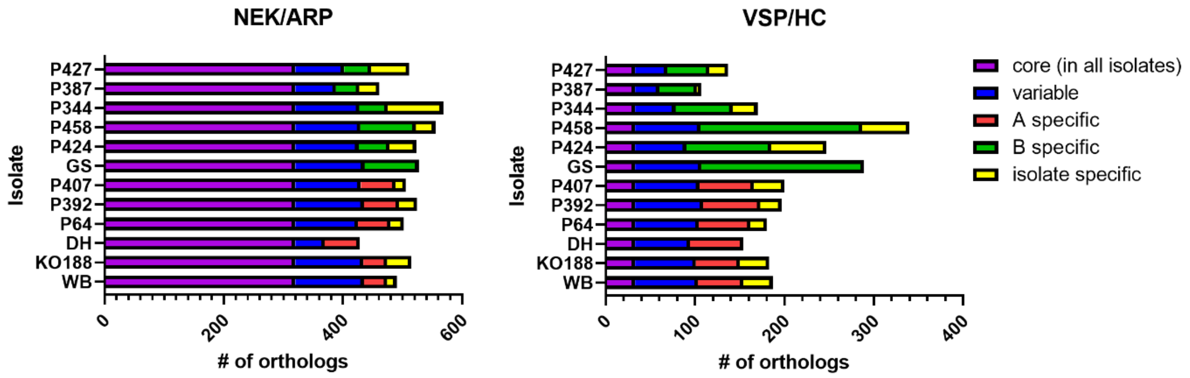

Fig. S5. Number of orthologous groups of known major *Giardia* protein families by isolate. Members of the major protein families NEK kinases and ankyrin repeat proteins (NEK/ARP) and variant surface proteins and high cysteine proteins (VSP/HC) are presented dependent of their representation in core, variable, assemblage or isolate specific group.

Supplementary Tables

Table S1: Description of raw reads data.

	PacBio RS II				Illumina			
Isolate	# Reads	# Bases	Coverage	G + C %	# Reads	# Bases	Coverage	G + C %
P387	102842	648696164	53.71	49.86	4442269	2097906188	173.69	50.00
P424	124644	787504514	65.20	48.35	5250784	2644896381	218.98	49.07
P458	162509	981429276	81.26	49.28	5274648	2081313725	172.32	49.33
KO188	141679	1210519804	100.22	49.31	21879616	6596972644	546.19	48.71
P344	135539	1120669167	92.78	50.08	3720699	1804529261	149.40	49.84
P427	130450	920451356	76.21	50.02	4297773	2007135321	166.18	49.93
P64	166919	1033700952	85.58	49.76	3604569	1649075978	136.53	49.55
P392	144685	1123045764	92.98	49.24	4585106	2216146465	183.48	49.70
P407	140018	1162695627	96.26	49.82	4578837	2129951997	176.35	50.04

Table S2: pairwise comparison of total size of syntenic regions between isolates and selected reference strains WB, DH, GSB

	KO188	P64	P392	P407	DH	WB	P387	P424	P458	P344	P427	GS
KO188	*NA	11408576	11271048	11257571	9680137	11321439	9461752	11195077	11097799	11076380	10376982	8773037
P64	NA	NA	11075607	11043022	9625354	11273487	9341607	10956016	10923327	10851766	10206467	8722946
P392	NA	NA	NA	11221745	9745130	11266405	9466010	11110352	10956090	10939908	10420906	8733265
P407	NA	NA	NA	NA	9658605	11416926	9346671	11087293	10928260	10889267	10443850	8722874
DH	NA	NA	NA	NA	NA	9543772	8284536	9299333	9163980	9279738	8677796	8123750
WB	NA	NA	NA	NA	NA	NA	9525850	11175935	11192672	11081527	10480607	8744160
P387	NA	NA	NA	NA	NA	NA	NA	9724713	9818296	9765981	9517578	7878873
P424	NA	NA	NA	NA	NA	NA	NA	NA	11662026	11381077	10838564	9252021
P458	NA	NA	NA	NA	NA	NA	NA	NA	NA	11325365	10740294	9453696
P344	NA	NA	NA	NA	NA	NA	NA	NA	NA	NA	10753271	8771711
P427	NA	NA	NA	NA	NA	NA	NA	NA	NA	NA	NA	8457231
GS	NA	NA	NA	NA	NA	NA	NA	NA	NA	NA	NA	NA

* NA = not analysed

1. Kozlov, A.M., et al., *RAxML-NG: a fast, scalable and user-friendly tool for maximum likelihood phylogenetic inference.* Bioinformatics, 2019. **35**(21): p. 4453-4455.
